# Anti-Colon Cancer Activity of Dietary Phytochemical Soyasaponin I and the Induction of Metabolic Shifts in HCT116

**DOI:** 10.3390/molecules27144382

**Published:** 2022-07-08

**Authors:** Xuewei Xia, Qianmin Lin, Ning Zhao, Jinzi Zeng, Jiajia Yang, Zhiyuan Liu, Riming Huang

**Affiliations:** 1 Guangdong Provincial Key Laboratory of Food Quality and Safety, College of Food Science, South China Agricultural University, Guangzhou 510642, China; xiaxuewei@stu.scau.edu.cn (X.X.); jasmine_linqm@163.com (Q.L.); zengjinzi1999@stu.scau.edu.cn (J.Z.); yangjiajia@stu.scau.edu.cn (J.Y.); aiden@stu.scau.edu.cn (Z.L.); 2 Graduate School, Guangzhou University of Chinese Medicine, Guangzhou 510006, China; 20211111548@stu.gzucm.edu.cn

**Keywords:** dietary phytochemicals, soyasaponin I, anti-colon cancer, network pharmacology, metabolomic analysis, molecular docking

## Abstract

Dietary phytochemicals play an important role in the prevention and treatment of colon cancer. It is reported that group B of soyasaponin, derived from dietary pulses, has anti-colonic effects on some colon cancer cell lines. However, it is uncertain which specific soybean saponins play a role. In our study, as one of the group B soyasaponin, the anti-colon cancer activity of soyasaponins I (SsI) was screened, and we found that it had the inhibitory effect of proliferation on colon cancer cell lines HCT116 (IC_50_ = 161.4 μM) and LoVo (IC_50_ = 180.5 μM), but no effect on HT29 between 0–200 μM. Then, nine potential targets of SsI on colon cancer were obtained by network pharmacology analysis. A total of 45 differential metabolites were identified by metabolomics analysis, and the KEGG pathway was mainly enriched in the pathways related to the absorption and metabolism of amino acids. Finally, molecular docking analysis predicted that SsI might dock with the protein of DNMT1, ERK1. The results indicated that the effect of SsI on HCT116 might be exerted by influencing amino acid metabolism and the estrogen signaling pathway. This study may provide the possibility for the application of SsI against colon cancer.

## 1. Introduction

Colon cancers are the third most common cancer disease around the world and are the second leading cause of cancer death, according to global cancer statistics for 2020 [1]. Early screening of colon cancer significantly decreases the death rate but is not widely available as it is limited by medical resources and personal willingness [2]. Thus, a prevention strategy to control the incidence of colon cancer is more attractive [3], especially with evidence that dietary phytochemicals may play a role in the prevention and treatment of colon cancer [4,5].

Soyasaponins are a major natural compound in many important dietary pulses whose structures are oleanane triterpenoids and are mainly classified into two groups, A and B [6,7]. Group B of soyasaponins is the most abundant group and is thought to contribute to the biological properties of pulses [6]. As a major constituent of group B, soyasaponin I (SsI) has been widely studied for its various biological activities [8]. It has been reported that SsI was found to be a kind of sialyltransferase inhibitor that did not affect growth, but inhibited migration in breast cancer [9], melanoma [10], and ovarian cancer [11]. In addition, it possesses other activities including anti-inflammatory effects [12,13], protecting rat liver cells [14], and improving the ability of neuroprotection and regeneration in memory-deficient model rats [15].

Although there have been some reports about the effect of group B soyasaponins on colon cancer, there are two opposite results. On the one hand, crude group B soyasaponins containing soyasaponins I, II, III, and IV were isolated from soybeans and could induce autophagy of human colon cancer HCT15 by inhibiting the Akt signaling pathway and enhancing the activity of ERK1/2, with an IC_50_ of about 100 p.p.m. [16,17]. On the other hand, purified group B soyasaponins including soyasaponins I, II, and III had no significant effect on human colon cancer HT29 in the concentration between 0–50 p.p.m.; SsI had the same result [18]. It is worth noting that HCT15 with mutant *KRAS* and HT29 with wild-type *KRAS* are two cell lines with different mutation types [19].

Since the colon cancer cell lines used in the aforementioned research are different, and there is no study on other group B soyasaponins against colon cancer, we selected the SsI with the most abundant constituent of group B soyasaponins to carry out our study. In our study, two colon cancer cell lines with mutant *KRAS* (HCT116 and LoVo), and one with wild-type *KRAS* (HT29) were screened for anti-colon cancer activity. It was found that SsI had no effect on HT29 cells in the concentration range of 0–200 μM but had an anti-colon cancer effect on HCT116 and LoVo with the lowest IC_50_ of HCT116. Based on this, we further speculated the possible mechanism of SsI against HCT116 through metabolomic analysis and molecular docking. This study may provide some evidence and possibilities for the application of SsI against colon cancer for further research.

## 2. Results

### 2.1. Effect of SsI on the Proliferation of Three Colon Cancer Cell Lines

**Figure 1:**
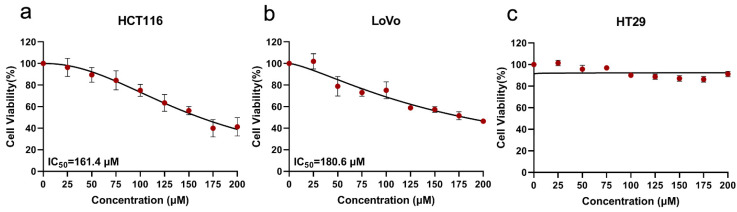
(**a**–**c**) The effect of SsI on colon cancer cell lines HCT116 (**a**), LoVo (**b**), and HT29 (**c**).

To investigate whether SsI has an anti-colon cancer effect, cell proliferation experiments were performed on colon cancer cell lines with mutant *KRAS* (HCT116 and LoVo) and wild-type *KRAS* (HT29) via CCK-8. As shown in Figure 1, SsI could inhibit the proliferation of HCT116 (IC_50_ = 161.4 μM) and LoVo (IC_50_ = 180.6 μM) with dose-dependence between the concentration of 0–200 μM. Meanwhile, there was no effect on HT29, as in previous studies [18,20]. The *KRAS* mutant colon cancer cells might be more sensitive to SsI.

### 2.2. Potential Targets and Pathways for SsI against Colon Cancer

**Figure 2:**
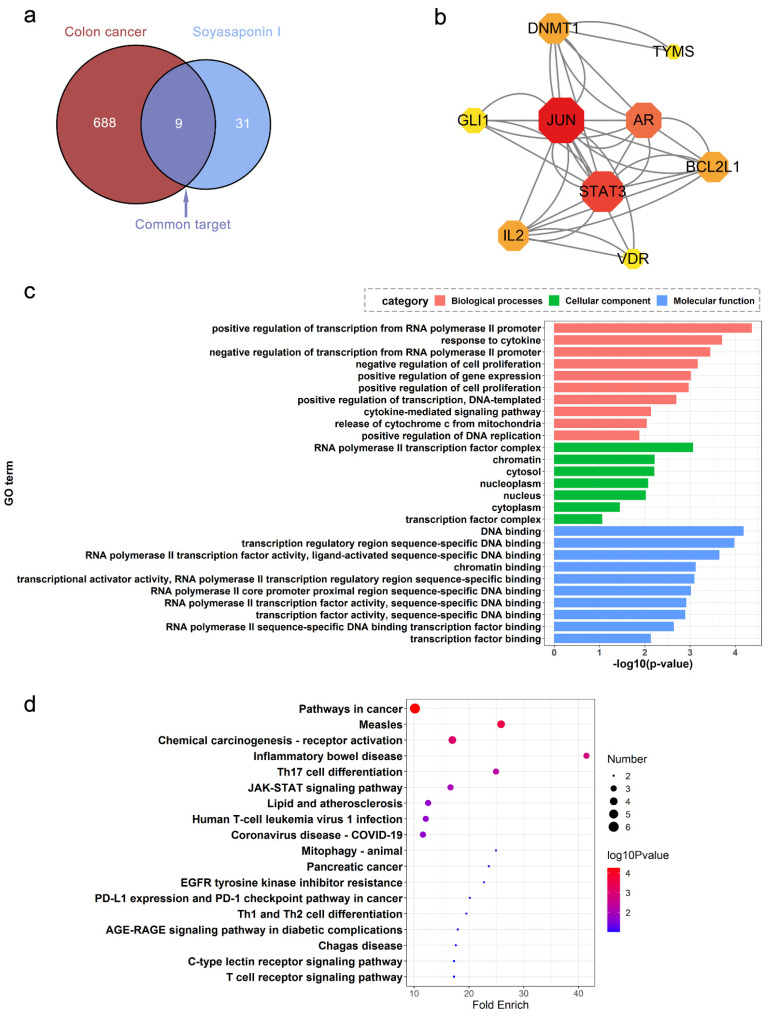
Network pharmacologic analysis of colon cancer by SsI. (**a**) Venn plots of common targets screened by the database. (**b**–**d**) PPI network (**b**), enriched GO term (**c**), and KEGG pathway of the 9 common targets for SsI and colon cancer.

A total of 39 potential targets of SsI and 697 colon cancer-relative targets were screened including nine common targets (Figure 2a). A PPI network of the nine common targets was further constructed to demonstrate the relationship between the targets (Figure 2b). The Gene Ontology (GO) enrichment analysis of common targets was annotated to 36 biological process (BP), 7 cellular component (CC), and 13 molecular function (MF). As shown in Figure 2c, the top 10 pathways were mainly related to cell proliferation and transcription. The Kyoto Encyclopedia of Genes and Genomes (KEGG) enrichment analysis suggested that four significantly enriched pathways were associated with cancer and inflammatory bowel disease, including “Pathways in cancer pathway”, “Measles”, “Chemical carcinogenesis—receptor activation”, and “Inflammatory bowel disease” (Figure 2d). It is speculated that SsI may affect these pathways and thus play an anticancer role.

### 2.3. Metabolomic Analysis for SsI against HCT116

**Figure 3:**
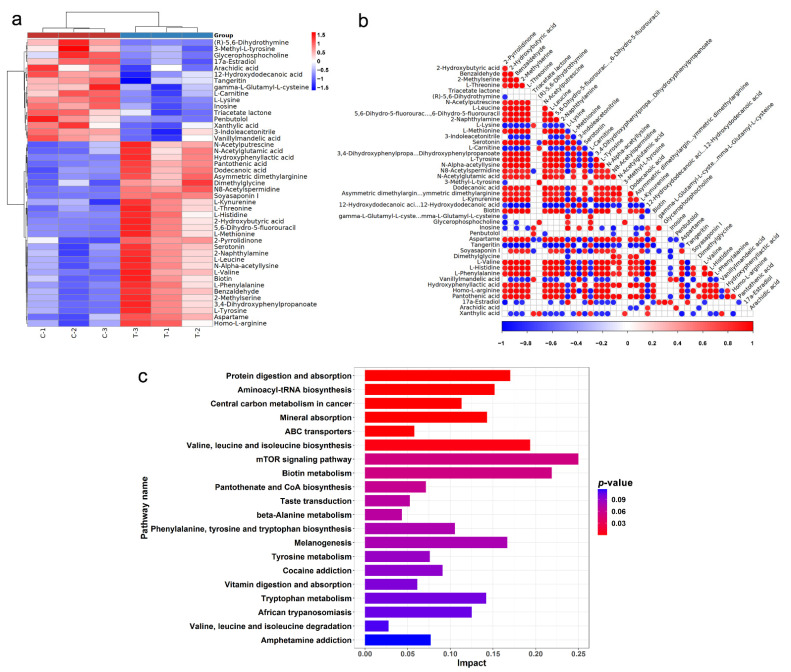
Metabolomics analysis of SsI against colon cancer cell lines HCT116. (**a**) Heatmap of DAMs between the control and treatment groups. (**b**) Pearson correlation heatmap. (**c**) The top 20 KEGG pathways sorted by *p*-value.

Since it had the lowest IC_50_, HCT116 was used to further experiment. Metabolomic analysis of HCT116 was performed to compare the metabolic differences after being treated with SsI. The SsI concentration of the treatment group was at approximately IC_50_ (160 mM) according to the result of the cell proliferation experiment. Multivariate statistical analysis by the OPLS-DA model demonstrated that each group was separated, which proved that the results were reliable (Figure S1). A total of 258 metabolites were identified by LC-MS/MS, of which 45 were defined as a differently accumulated metabolite (DAM) by *p*-value < 0.05, VIP > 1 (Table S1). The positive and negative values of Log_2_(FC) represented the up-regulation or down-regulation of DAM, and the absolute value represents the change degree. The top 20 DAMs by change degree are listed in Table 1. Compared with the control group, 29 DAMs were up-regulated and 16 DAMs were down-regulated (Figure 3a). The correlation between each DAM was analyzed by calculating the Pearson correlation coefficient between pairs of DAMs (Figure 3b).

The KEGG enrichment analysis showed that the 45 DAMs were involved in 55 pathways, among which 6 pathways were significantly enriched with *p*-value < 0.05 (Figure 3c and Table S2). The enriched pathways were mainly related to the amino acid, including “Protein digestion and absorption”, “Aminoacyl-tRNA biosynthesis”, “Central carbon metabolism in cancer”, “Mineral absorption”, “ABC transporters”, and “Valine, leucine, and isoleucine biosynthesis”.

### 2.4. The Prediction of Molecular Docking

**Figure 4:**
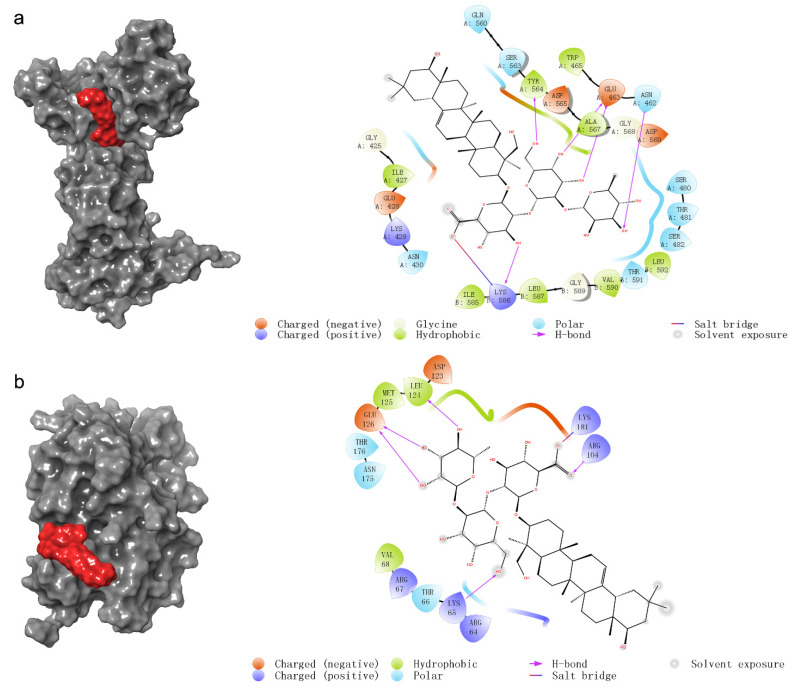
(**a**,**b**) Prediction of molecular docking between SsI and protein DNMT1 (**a**) and ERK1 (**b**).

To further confirm the possible effect of SsI on HCT116, we predicted the docking mode between SsI and nine common target proteins of network pharmacology. In addition, due to the attention to the estrogen signaling pathway, we also conducted molecular docking analysis on key proteins of the estrogen signaling pathway. The molecular docking results are summarized in Table 2. The docking modes with docking scores less than −4 are shown in Figure 4. The prediction of molecular docking analysis suggested that SsI might bond to DNMT1 with a docking score of −5.314 by forming five hydrogen bonds and a salt bridge (Figure 4a). Five hydrogen bonds and a salt bridge also occurred between SsI and ERK1 but with a lower docking score of −4.313 (Figure 4b). The results indicated that SsI might be bound to DNMT1 and ERK1 to inhibit the proliferation of HCT116. However, there may be no direct effect between SsI and ER.

## 3. Discussion

With a very special demand for nutrition, cancer cells increased multiple metabolic processes and overconsumed nutrients in order to meet the energy and biosynthesis required for growth [21]. In past studies, it had been found that the consumption of certain amino acids, such as asparagine, arginine, and methionine, could be applied to treat amino acid-dependent cancers [22]. In addition, the degradation of lysine could also inhibit the growth of various cancer cells [23]. In the metabolomic analysis of our study, seven essential amino acids were contained in the DAMs and lysine was significantly down-regulated. The enriched KEGG pathway was mainly related to the absorption and metabolism of amino acids. It suggested that SsI may affect the amino acid metabolism of HCT116 cells, resulting in lysine consumption.

Meanwhile, what caught our attention was the significant downregulation of 17α-estradiol (α-E_2_) in the DAMs. Chang et al. [24] reported that SsI with an estrogenic effect could promote the proliferation and activate the transcription of estrogen-responsive genes c-*fos* and *pS2* on breast cancer cell line MCF-7. The estrogen signaling pathway associated with α-E_2_ also included the protein encoded by *KRAS*. While the gene *KRAS* is the mutation site of two SsI-sensitive cell lines HCT116 and LoVo. It is speculated that SsI might influence the estrogen signaling pathway to play an anticancer role. This brought us to the role of estrogen and estrogen receptors in cancer.

Estrogen could activate downstream signaling pathways through estrogen receptors (ER), leading to cell proliferation or apoptosis [25]. This difference in effects mainly depended on which types of estrogen receptor received the signal. With stimulation by estrogen, ER-α increased the proliferation of cancer cells, while ER-β inhibited the proliferation of cancer cells and led to apoptosis [26,27]. ER-β was expressed commonly in intestinal cells, with very low or no expression of ERα, even in colon cancer cells [28,29,30]. HCT116 cells were found to express ER-β but not ERα (ERα−/ERβ+) [31]. Therefore, the inhibition of HCT116 proliferation by SsI might be through its estrogen effect, to stimulate ER-β and then activate the tumor suppressor genes.

A similar effect occurred in soyasapogenol, which is structurally similar to SsI. Rowlands et al. [32] found that soyasapogenol A had estrogenic activity and could inhibit the proliferation of breast cancer cell line MDA-MB-231 (ERα−/ERβ+), but it was uncertain whether this effect was through direct or indirect activation of ER. Thus, we subsequently predicted the combination of SsI and ER by Maestro software (v11.8). The predicted results showed that there was no docking mode between SsI and ER-β, and the same with ER-α.

As for ERK1/2 in the estrogen signaling pathway, the study had shown that crude extracts of group B soyasaponin can improve the activity of ERK1/2 to induce autophagy of human colon cancer HCT15 [16,17]. Our molecular docking analysis predicted that SsI docking to Lys65, Arg104, Glu126, Leu124, and Lys181 of ERK1 occupied the backside binding site of ERK1 [33]. SsI might improve the activity of ERK1 by this active site.

Collectively, we speculated that SsI might affect the estrogen signaling pathway by indirectly affecting ER, or increasing the activity of ERK1, to activate the downstream signaling pathway and inhibit the proliferation of cancer cells. As for the down-regulation of α-E_2_, we speculated that the estrogen effect of SsI might lead to some kind of negative feedback regulating α-E_2_, one of the estrogens [34].

In addition, DNMT1 had the best docking score by molecular docking prediction of nine common targets obtained from network pharmacology. DNMT1 and ZNF304 could form the complex required for tumor suppressor gene transcriptional silencing to protect colon cancer cells [35,36]. SsI may bind to DNMT1, preventing the formation of this complex and restoring the transcription of tumor suppressor genes.

Considering the anti-colon cancer activity of SsI, it is meaningful to consider its bioavailability. However, there are only a few studies on the bioavailability of SsI, with varying results. Two studies by Hu et al. [37,38] preliminarily demonstrated the biological effects of SsI ingestion. First of all, in the in vitro fecal fermentation system, SsI could be transformed into soyasaponol B through human gut microbial [37]. Then, female volunteers ingested food containing 436 μmol group B soyasaponins composed of 233.6 μmol SsI. SsI was not detected in feces and urine, but a total of 36.3 ± 10.2 μmol soyasaponol B was extracted from feces [38]. In addition, the result of transepithelial kinetics showed that SsI can across the Caco-2 monolayer with the highest apparent coefficient (*P_app_*) of 3.6 ± 0.5 × 10^−6^ cm/s at the concentration of 0.5 mM. Their research confirmed the conversion of SsI to soyasaponol B by human gut microbial in vitro and in vivo, but did not test for the presence of SsI in plasma. Other studies by Neacsu et al. [39] showed that after volunteers continued to eat soybean products as meals for 7 days, SsI content was 0.26 to 1.19 mg in 100 g fresh fecal sample, and SsI concentration in plasma was the highest at 24.5 ± 15.5 ng/mL at 3 h postprandial. The presence of SsI in plasma indicated that SsI can be absorbed by the body, but no specific content of SsI in these foods was stated. The differences in fecal SsI content between the result from Hu et al. and Neacsu et al. can be explained in part by the possibility of individual differences in human gut microbial metabolism or absorption of SsI.

Compared with our result, the highest SsI concentration in plasma was 24.5 ± 15.5 ng/mL (about 26 ± 16.4 nM) by Neacsu et al. [39], which was extremely low. Therefore, it may be possible to consider direct absorption of SsI at the site of colon cancer to play a role. The finding by Sagratini et al. [40] in the bioaccessibility of SsI extracted from cooked lentils can also give us some insight. The bioaccessibility values of SsI ranged from 8.9 to 10.6% in the duodenum compartment and from 0.08 to 0.27% in the colon compartment using the simulated gastrointestinal in vitro digestion. Surprisingly, the prolonged cooking time of lentils enhances bioaccessibility at the intestinal level. Perhaps future research should focus on allowing SsI to reach the colon, for example, by delaying the degradation of SSI by gut microbial.

## 4. Materials and Methods

### 4.1. Stock Solution of SsI

The pure compound of soyasaponin I (SsI, CAS: 51330-27-9) was acquired from Nanjing Plant Origin Biological Technology Co., Ltd. (Nanjing, China). SsI was dissolved in dimethyl sulfoxide as a stock solution (200 mM) and stored at −20 °C.

### 4.2. Cell Culture

The human colon cancer cell lines HCT116, LoVo, and HT29 were obtained from Procell Life Science & Technology Co., Ltd. (Wuhan, China) with the short tandem repeat (STR) authentication. All experiments have used the cells in generations from 10 to 20. The cells were cultured in an incubator with 5% CO_2_ at 37 °C. The medium used was DMEM with 10% (*v*/*v*) FBS and 1% (*v*/*v*) penicillin–streptomycin and replaced every 2 days. All the reagents were purchased from Thermo Fisher Scientific (Waltham, MA, USA).

### 4.3. Evaluation of Cell Proliferation

The cell proliferation of HCT116, LoVo, and HT29 was measured by Cell Counting Kit-8 (CCK-8, dib, Guangzhou, China). The experiments were carried out with six replicates. Cells were seeded into 96-well plates with the amount of 5000 cells per well. After 24 h cultured, the fresh medium with SsI at the final concentration of 0, 25, 50, 75, 100, 125, 150, 175, and 200 μM was prepared by diluting SsI stock solution (200 mM), and used to replace the old medium. After an additional 24 h of SsI treatment, 10 μL CCK-8 reagent was added to each well and incubated at 37 °C for 2 h, then the absorbance value was detected at 450 nm. The IC_50_ of SsI against colon cancer cells was analyzed and plotted using GraphPad Prism software (v8.0.2).

### 4.4. Network Pharmacologic Analysis

#### 4.4.1. Screen of Interaction Targets

The compound-related targets of SsI were searched on the database of Traditional Chinese Medicine Systems Pharmacology (TCMSP, https://old.tcmsp-e.com/), Integrative Pharmacology-based Research Platform of Traditional Chinese Medicine (TCMIP, http://www.tcmip.cn/), SwissTargetPrediction (http://www.swisstargetprediction.ch/), and TargetNet (http://targetnet.scbdd.com/). The disease-related target of colon cancer was searched on PharmGkb (https://www.pharmgkb.org/), GeneCards^®^ (https://www.genecards.org/), Online Mendelian Inheritance in Man^®^ (OMIM^®^, https://www.omim.org/), Comparative Toxicogenomics Database (CTD, https://ctdbase.org/), and Therapeutic Target Database (TTD, http://db.idrblab.net/ttd/). The Uniprot database (https://www.uniprot.org/) was applied to unify the symbols of target proteins and identified the common interaction targets. The above databases were accessed on 21 February 2022.

#### 4.4.2. Enrichment Analysis and Construction of the Compound-Target-Pathway Network

The Gene Ontology (GO) and Kyoto Encyclopedia of Genes and Genomes (KEGG) enrichment analyses were performed via the Database for Annotation, Visualization, and Integrated Discovery v6.8 (DAVID v6.8) [41]. The protein–protein interaction (PPI) networks were built via STRING v11.5 [42]. The common interaction targets were uploaded to STRING with the parameter setting of Required score to highest confidence (0.900) and FDR stringency to medium (5 percent), and disconnected nodes were hidden in the network. Based on the results of the KEGG enrichment analysis and PPI network, the compound-target-pathway (C-T-P) networks were constructed by Cytoscape software (v3.9.0).

MetaboAnalyst v5.0. https://www.metaboanalyst.ca/ (accessed on 17 March 2022).

### 4.5. Metabolomic Analysis

The metabolomic analysis was performed based on previous methods [43,44,45]. The experiment contained the control group (0 μM) and treatment group (160 μM) with three replicates treated with different concentrations of SsI. The cells were collected after 24 h treated and immediately quenched with liquid nitrogen for subsequent experiments.

All cell samples were mixed with 100 mg glass beads and 1000 µL water solution of 40% acetonitrile and 40% methanol (*v*/*v*). Cells were broken with the pulses of vortexing for 30 s, then were ground three times at 55 Hz for 2 min, keeping the cells on liquid nitrogen for 5 min between each grinding. After cell crushing, centrifugation was performed at 12,000 rpm and 4 °C for 10 min, then all the supernatant was concentrated dried. The sample was redissolved by 300 µL acetonitrile solution with 0.04% 2-Amino-3-(2-chloro-phenyl)-propionic acid and 0.09% formic acid (*v*/*v*). Then, the redissolved sample was filtered by 0.22 µm membrane and transferred into the detection bottle.

The liquid chromatography analysis was performed on a Vanquish UHPLC System (Thermo Fisher Scientific, Waltham, MA, USA) with the column of ACQUITY UPLC ^®^ HSS T3 (150 × 2.1 mm, 1.8 µm) (Waters, Milford, MA, USA). The column temperature was set at 40 °C, the flow rate was 0.25 mL/min, and the injection volume was 2 µL. The mobile phase consisted of acetonitrile with 0.1% formic acid (*v*/*v*) (A) and water solution of 0.1% formic acid (*v*/*v*) (B) in positive ion (ESI+) mode, acetonitrile (C) and 5 mM ammonium formate (D) for negative ion (ESI-) mode. The procedure of gradient elution was performed as shown in Tables S3 and S4.

Mass spectrometric (MS) detection of metabolites was performed on Orbitrap Exploris 120 (Thermo Fisher Scientific, Waltham, MA, USA) with ESI ion source. Simultaneous MS1 and MS/MS (Full MS-ddMS2 mode, data-dependent MS/MS) acquisition was used. The setting parameters were shown in Table S5.

Build of the orthogonal partial least-squares discriminant analysis (OPLS-DA) models were performed by using the ropls package in R language (v1.3.959), and the variable importance in the projection (VIP) value was obtained from it. The *p*-value was produced by the Wilcoxon–Mann–Whitney test. The metabolites with VIP value > 1 and *p*-value < 0.05 were considered to be statistically significant and labeled as differential accumulated metabolites (DAMs). DAMs were subjected to KEGG pathway analysis by MetaboAnalyst v5.0 [46] and selected pathways with *p*-values < 0.05.

### 4.6. Molecular Docking

The molecular docking was conducted by Schrodinger Maestro software (v11.8) to predict the possible binding mode between SsI and proteins. The structure of SsI was downloaded from PubChem (https://pubchem.ncbi.nlm.nih.gov/, Compound CID: 122097, accessed on 7 April 2022). The three-dimensional structure of the target protein was available from the UniProt website (https://www.uniprot.org/, accessed on 7 April 2022). Further protein preparation removed all water molecules and added polar hydrogen. An RMSD less than 2.0 was considered a reliable docking [47]. Finally, the model with the lower docking score was considered better.

### 4.7. Statistical Analysis

The mathematical-statistical analysis of the data was conducted via SPSS software v24.0. The data are expressed as mean ± SD. One-way analysis of variance (ANOVA) was used for comparison between the different groups. The *p*-values less than 0.05 were considered to be statistically significant.

## 5. Conclusions

This study suggested that SsI may regulate seven essential amino acids and impact amino acid metabolism, and the downregulation of lysine may indicate the occurrence of lysine consumption. The estrogen signaling pathway might be a key pathway for SsI against colon cancer, which may stimulate ER indirectly or affect downstream pathways through ERK1. Moreover, DNMT1 may also be its potential direct target. Since some of the results were predicted by computer, these potential possibilities needed to be further verified by experiments. However, this study provides some evidence for group B soyasaponins against colon cancer.

## Figures and Tables

**Table 1:** The top 20 DAMs with the largest absolute value of log_2_(FC).

Name	KEGG Entry	log_2_(FC)	*p*-Value	VIP	Trend
Soyasaponin I	C08983	15.52	0	1.82	up
Dodecanoic acid	C02679	2.24	0.0036	1.74	up
N8-Acetylspermidine	C01029	1.21	0.0001	1.80	up
2-Hydroxybutyric acid	C05984	0.99	0.0433	1.68	up
L-Methionine	C00073	0.99	0.0402	1.69	up
5,6-Dihydro-5-fluorouracil	C16630	0.98	0.0408	1.68	up
Pantothenic acid	C00864	0.97	0.0013	1.83	up
2-Pyrrolidinone	C11118	0.92	0.0132	1.65	up
Hydroxyphenyllactic acid	C03672	0.86	0.0349	1.76	up
Biotin	C00120	0.83	0.0205	1.62	up
2-Naphthylamine	C02227	0.79	0.0165	1.64	up
Xanthylic acid	C00655	−0.8	0.0383	1.60	down
Triacetate lactone	C02752	−0.84	0.0189	1.60	down
3-Methyl-L-tyrosine	C20800	−0.86	0.0395	1.52	down
Vanillylmandelic acid	C05584	−1.31	0.0259	1.64	down
Penbutolol	C07416	−1.41	0.0121	1.65	down
L-Lysine	C00047	−1.54	0.0003	1.79	down
Arachidic acid	C06425	−1.69	0.0465	1.53	down
(R)-5,6-Dihydrothymine	C21028	−3.00	0.0283	1.72	down
17a-Estradiol	C02537	−3.13	0.0047	1.78	down

**Table 2:** The docking score table of molecular docking for SsI and proteins.

Category	Protein	PDB ID	Docking Score *	Amino Acid Residues
common target proteins of network pharmacology	DNMT1	3epz	−5.314	ASN462, GLU463, TYR564, LYS586
	STAT3	6njs	−3.863	ARG609, SER611, GLU612, SER613
	VDR	1db1	−3.706	SER265, GLU395, ARG402
	BCL2L1	1bxl	−3.396	ARG34, GLU36, GLU44
	IL2	1irl	−3.192	THR3, GLN13, GLU95
	GLI1	2gli	-	-
	AR	1t65	-	-
	JUN	1s9k	-	-
	TYMS	1hvy	-	-
key proteins of the estrogen signaling pathway	ERK1	2zoq	−4.313	Lys65, Arg104, Glu126, Leu124, Lys181
	ERK2	4qp6	−3.917	ILE86, ASP106
	ESR1(ER-α)	1a52	-	-
	ESR2(ER-β)	1qkm	-	-

* Symbol “-” represents that no docking mode is generated after analyses.

## Data Availability

The data presented in this study are available in Supplementary Materials.

## Supplementary Materials

The following supporting information can be downloaded at: https://www.mdpi.com/article/10.3390/molecules27144382/s1, Figure S1: OPLS-DA score plot of metabolomic analysis in negative ion (ESI-) mode (a) and positive ion (ESI+) mode (b); Table S1: The results of metabolite difference analysis; Table S2: The results of KEGG enrichment analysis; Table S3: The gradient elution procedure of liquid chromatography in ESI+ mode; Table S4: The gradient elution procedure of liquid chromatography ESI- mode; Table S5: Mass spectrum conditions.
