# Supplementary material for: Anti-Colon Cancer Activity of Dietary Phytochemical Soyasaponin I and the Induction of Metabolic Shifts in HCT116
Source: Molecules. 2022 Jul 8;27(14):4382. doi: 10.3390/molecules27144382 (PMC9316303; doi:10.3390/molecules27144382)
Supplement: Supplementary file 1 [file molecules-27-04382-s001.zip › molecules-1780939-supplementary.pdf]

Supplementary Materials

Figures

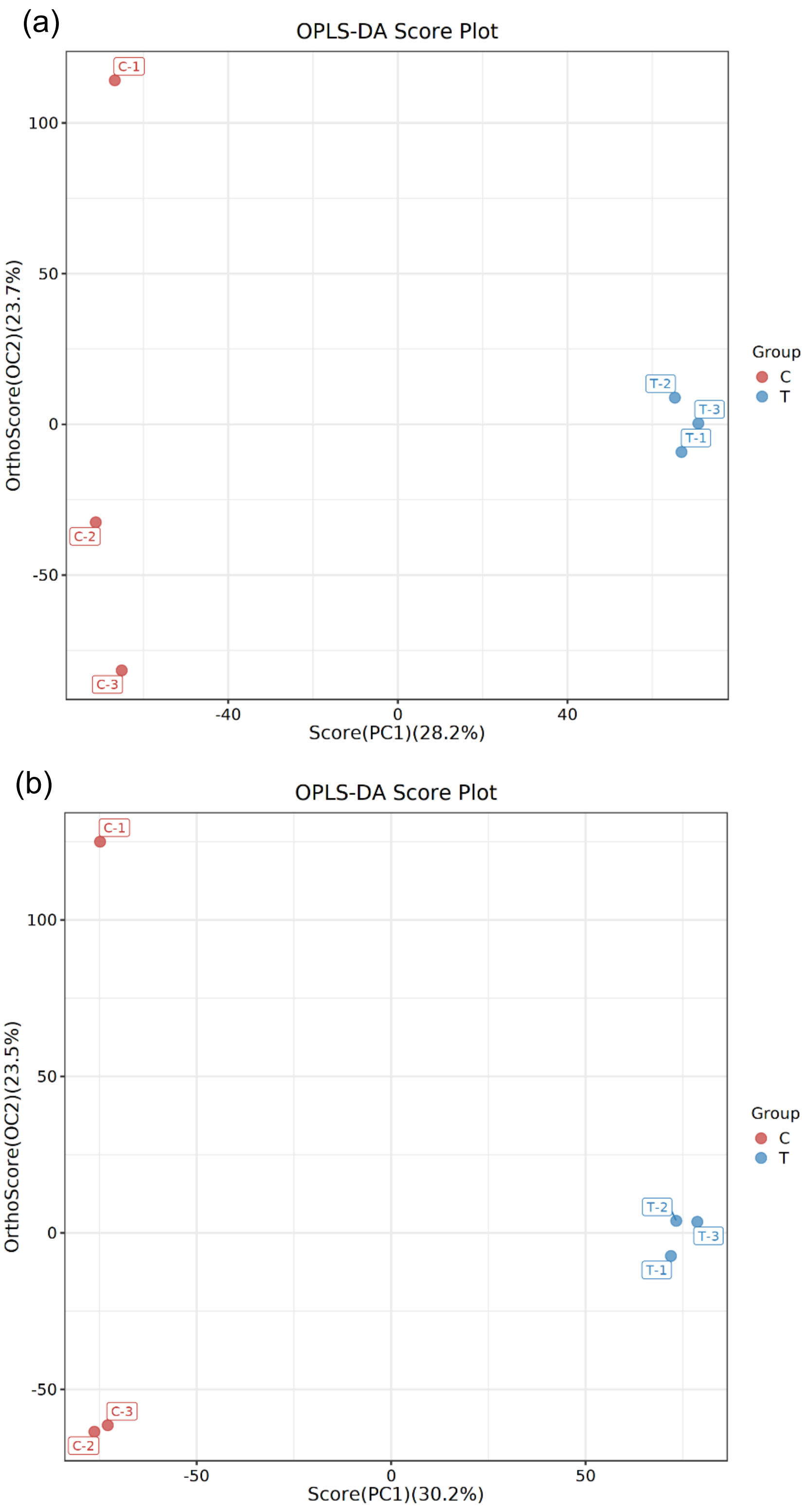

Figure S1. OPLS-DA score plot of metabolomic analysis in negative ion (ESI-) mode (a) and positive ion (ESI+) mode (b).

Tables

Table S1. The results of metabolite difference analysis.

Name	KEGG entry	FC	log2FC	P.value	VIP	Trend
Soyasaponin I	C08983	46951.68	15.52	0.0000	1.82	up
N8-Acetylspermidine	C01029	2.31	1.21	0.0001	1.80	up
L-Lysine	C00047	0.34	-1.54	0.0003	1.79	down
Pantothenic acid	C00864	1.96	0.97	0.0013	1.83	up
Inosine	C00294	0.70	-0.51	0.0018	1.74	down
Asymmetric dimethylarginine	C03626	1.27	0.35	0.0022	1.76	up
Dodecanoic acid	C02679	4.71	2.24	0.0036	1.74	up
17a-Estradiol	C02537	0.11	-3.13	0.0047	1.78	down
Aspartame	C11045	1.50	0.58	0.0050	1.73	up
Benzaldehyde	C00261	1.58	0.66	0.0064	1.71	up
L-Valine	C00183	1.71	0.77	0.0078	1.76	up
L-Tyrosine	C00082	1.65	0.72	0.0086	1.69	up
L-Carnitine	C00318	0.72	-0.48	0.0090	1.67	down
L-Phenylalanine	C00079	1.62	0.70	0.0092	1.75	up
Serotonin	C00780	1.61	0.69	0.0096	1.69	up
L-Threonine	C00188	1.50	0.59	0.0098	1.68	up
3,4-Dihydroxyphenylpropanoate	C10447	1.63	0.71	0.0108	1.68	up
L-Leucine	C00123	1.53	0.61	0.0117	1.67	up
Dimethylglycine	C01026	1.49	0.58	0.0117	1.69	up
Penbutolol	C07416	0.38	-1.41	0.0121	1.65	down
2-Methylserine	C02115	1.62	0.70	0.0124	1.67	up
N-Alpha-acetyllysine	C12989	1.62	0.70	0.0127	1.67	up
N-Acetylputrescine	C02714	1.24	0.31	0.0130	1.67	up
2-Pyrrolidinone	C11118	1.89	0.92	0.0132	1.65	up
N-Acetylglutamic acid	C00624	1.46	0.54	0.0143	1.66	up
L-Kynurenine	C00328	1.34	0.42	0.0156	1.64	up
2-Naphthylamine	C02227	1.73	0.79	0.0165	1.64	up
12-Hydroxydodecanoic acid	C08317	0.90	-0.15	0.0176	1.64	down
Triacetate lactone	C02752	0.56	-0.84	0.0189	1.60	down
Biotin	C00120	1.78	0.83	0.0205	1.62	up
Homo-L-arginine	C01924	1.41	0.49	0.0226	1.67	up
Vanillylmandelic acid	C05584	0.40	-1.31	0.0259	1.64	down
(R)-5,6-Dihydrothymine	C21028	0.12	-3.00	0.0283	1.72	down
gamma-L-Glutamyl-L-cysteine	C00669	0.70	-0.50	0.0328	1.52	down
Hydroxyphenyllactic acid	C03672	1.82	0.86	0.0349	1.76	up
Xanthylic acid	C00655	0.58	-0.80	0.0383	1.60	down
3-Methyl-L-tyrosine	C20800	0.55	-0.86	0.0395	1.52	down
L-Methionine	C00073	1.99	0.99	0.0402	1.69	up
Tangeritin	C10190	0.70	-0.51	0.0405	1.53	down
5,6-Dihydro-5-fluorouracil	C16630	1.98	0.98	0.0408	1.68	up

2-Hydroxybutyric acid	C05984	1.99	0.99	0.0433	1.68	up
Arachidic acid	C06425	0.31	-1.69	0.0465	1.53	down
3-Indoleacetonitrile	C02938	0.66	-0.60	0.0492	1.48	down
L-Histidine	C00135	1.60	0.67	0.0499	1.72	up
Glycerophosphocholine	C00670	0.78	-0.36	0.0499	1.48	down
Aminohydroquinone	C14604	0.68	-0.56	0.0503	1.46	none
N-Carbamoylputrescine	C00436	4.44	2.15	0.0521	1.66	none
Pseudouridine	C02067	1.58	0.66	0.0554	1.52	none
Spermidine	C00315	2.16	1.11	0.0578	1.46	none
S-Adenosyl-4-methylthio-2-oxobutanoate	C04425	0.33	-1.58	0.0683	1.41	none
Guanosine	C00387	0.72	-0.47	0.0715	1.41	none
Trigonelline	C01004	2.75	1.46	0.0750	1.38	none
3-Androstanol	C15638	0.91	-0.14	0.0803	1.40	none
Methyl jasmonate	C11512	0.13	-2.97	0.0805	1.56	none
Oxoglutaric acid	C00026	2.81	1.49	0.0813	1.54	none
myo-Inositol	C00137	0.19	-2.42	0.0893	1.41	none
Phosphorylcholine	C00588	0.72	-0.47	0.0952	1.32	none
S-Adenosylhomocysteine	C00021	1.88	0.91	0.0985	1.34	none
Rimantadine	C07236	0.85	-0.23	0.1015	1.34	none
11-Dehydrocorticosterone	C05490	0.49	-1.02	0.1019	1.35	none
Taurine	C00245	1.32	0.40	0.1030	1.33	none
Niacinamide	C00153	0.15	-2.73	0.1035	1.33	none
N-Acetylornithine	C00437	1.18	0.24	0.1084	1.34	none
Sphingosine	C00319	0.21	-2.23	0.1121	1.30	none
N-Acetyl-L-aspartic acid	C01042	1.35	0.43	0.1137	1.31	none
4-Guanidinobutanoic acid	C01035	1.19	0.25	0.1151	1.30	none
Vanillic acid	C06672	4.30	2.10	0.1160	1.26	none
Phenyl acetate	C00548	0.46	-1.12	0.1217	1.28	none
1D-Myo-inositol 1,4-bisphosphate	C01220	5.41	2.44	0.1228	1.51	none
Sorbitol	C00794	7.14	2.84	0.1233	1.25	none
5-Guanidino-3-methyl-2-oxopentanoate	C20234	1.32	0.40	0.1243	1.30	none
N-Acetyl-D-glucosamine	C00140	1.25	0.32	0.1282	1.25	none
Guanine	C00242	0.76	-0.40	0.1297	1.25	none
dTMP	C00364	0.23	-2.09	0.1339	1.26	none
Retinoyl b-glucuronide	C11061	0.21	-2.22	0.1353	1.25	none
Ascorbate	C00072	0.92	-0.11	0.1363	1.27	none
4-Quinolinecarboxylic acid	C06414	0.81	-0.31	0.1379	1.29	none
IMP	C00130	0.44	-1.17	0.1412	1.29	none
Creatinine	C00791	0.25	-1.99	0.1485	1.20	none
Protoporphyrinogen IX	C01079	0.23	-2.12	0.1505	1.22	none
Xanthine	C00385	1.22	0.29	0.1529	1.23	none
L-Methionine S-oxide	C02989	1.19	0.25	0.1552	1.18	none
Oxalacetic acid	C00036	0.87	-0.20	0.1563	1.23	none
Azelaic acid	C08261	1.46	0.54	0.1573	1.25	none

Glycyl-leucine	C02155	1.61	0.68	0.1701	1.13	none
5-Aminopentanoic acid	C00431	0.26	-1.93	0.1725	1.17	none
(3S)-3,6-Diaminohexanoate	C01142	0.93	-0.10	0.1738	1.13	none
Isocitric acid	C00311	1.97	0.98	0.1831	1.17	none
4,5-Dihydroorotic acid	C00337	1.85	0.89	0.1883	1.13	none
Creatine	C00300	0.87	-0.19	0.1905	1.09	none
allopurinol	C06816	1.71	0.77	0.1906	1.12	none
GMP	C00144	0.65	-0.61	0.1955	1.13	none
Aflatoxin B1	C06800	0.79	-0.33	0.2015	1.15	none
(S)-4-Hydroxymandelate	C03198	0.42	-1.25	0.2017	1.10	none
Mesaconate	C01732	1.20	0.27	0.2114	1.11	none
Deoxyuridine	C00526	0.93	-0.10	0.2122	1.11	none
Uridine diphosphate glucuronic acid	C00167	0.63	-0.66	0.2128	1.16	none
trans-trans-Muconic acid	C02480	0.61	-0.71	0.2133	1.11	none
Oxoadipic acid	C00322	1.26	0.33	0.2160	1.15	none
Acetylcholine	C01996	0.91	-0.13	0.2168	1.05	none
S-Glutathionyl-L-cysteine	C05526	0.71	-0.49	0.2211	1.05	none
Rosmarinic acid	C01850	0.82	-0.28	0.2224	1.06	none
Hypoxanthine	C00262	0.40	-1.33	0.2246	1.14	none
Lidocaine	C07073	0.91	-0.14	0.2280	1.21	none
Porphobilinogen	C00931	0.90	-0.15	0.2335	1.07	none
4-Acetamidobutanoic acid	C02946	1.32	0.41	0.2351	1.11	none
Neocembrene	C09140	0.89	-0.16	0.2371	1.06	none
Acetylphosphate	C00227	0.65	-0.61	0.2371	1.02	none
D-Galactose	C00124	0.39	-1.35	0.2445	1.10	none
L-Asparagine	C00152	0.62	-0.68	0.2476	1.04	none
Dihydroxyacetone phosphate	C00111	1.11	0.15	0.2563	1.00	none
Mirtazapine	C07570	0.91	-0.13	0.2587	1.02	none
2-Iminobutanoate	C20905	1.69	0.76	0.2690	0.99	none
Fumaric acid	C00122	1.73	0.79	0.2693	1.11	none
Fructose-1P	C10906	0.42	-1.25	0.2820	1.03	none
ADP	C00008	1.97	0.98	0.2867	0.97	none
Cyclic ADP-ribose	C13050	1.09	0.12	0.2881	0.98	none
Xanthosine	C01762	0.87	-0.20	0.2917	0.97	none
Dihydrouracil	C00429	0.78	-0.36	0.2917	0.98	none
L-Erythrulose	C02045	1.99	0.99	0.3053	0.96	none
Pyridoxine	C00314	0.89	-0.17	0.3082	0.87	none
16-Hydroxy hexadecanoic acid	C18218	0.57	-0.80	0.3114	0.93	none
L-2,4-diaminobutyric acid	C03283	0.83	-0.26	0.3135	0.89	none
L-Glutamic acid	C00025	0.83	-0.27	0.3194	0.87	none
UDP-N-acetyl-D-mannosamine	C01170	0.70	-0.52	0.3222	0.91	none
N-Acetyl-D-galactosamine	C01132	1.19	0.26	0.3290	0.87	none
L-Homophenylalanine	C17235	1.32	0.40	0.3417	0.94	none
CDP	C00112	1.39	0.48	0.3454	0.84	none

L-Aspartic acid	C00049	0.59	-0.75	0.3550	0.94	none
2-Methyl-3-oxopropanoic acid	C00349	1.35	0.43	0.3600	0.82	none
Beta-Leucine	C02486	0.67	-0.57	0.3654	0.83	none
Palmitoyl-L-carnitine	C02990	0.41	-1.28	0.3658	0.83	none
3-(2-Hydroxyphenyl)propanoic acid	C01198	1.06	0.09	0.3762	0.82	none
3-Methoxyanthranilate	C05831	1.20	0.27	0.3848	0.77	none
Citramalic acid	C00815	6.61	2.72	0.3849	0.81	none
3-Dehydroshikimate	C02637	1.40	0.49	0.3864	0.81	none
4-Pyridoxic acid	C00847	0.70	-0.52	0.3911	0.87	none
3-Hydroxymethylglutaric acid	C03761	3.03	1.60	0.3942	0.81	none
Ursodeoxycholic acid	C07880	0.96	-0.05	0.3972	0.82	none
Epsilon-caprolactam	C06593	1.12	0.17	0.3987	0.76	none
Fluorouracil	C07649	0.52	-0.96	0.3991	0.84	none
Undecanoic acid	C17715	1.64	0.71	0.4010	0.79	none
Catechol	C00090	2.42	1.27	0.4054	0.77	none
3-Methylthiopropionic acid	C08276	0.54	-0.90	0.4070	0.81	none
Picolinic acid	C10164	1.41	0.50	0.4143	0.72	none
Gamma-Linolenic acid	C06426	0.88	-0.18	0.4153	0.79	none
Butyryl-L-carnitine	C02862	1.09	0.13	0.4246	0.76	none
Phloroglucinol	C02183	0.61	-0.70	0.4250	0.75	none
Caffeate	C01197	0.44	-1.19	0.4290	0.78	none
Adenosine	C00212	1.07	0.10	0.4316	0.72	none
L-2-Hydroxyglutaric acid	C03196	2.31	1.21	0.4355	0.73	none
Pyrrolidonecarboxylic acid	C02237	1.75	0.81	0.4384	0.69	none
Nicotine	C00745	0.57	-0.82	0.4400	0.70	none
Ethirimol	C18828	0.91	-0.14	0.4409	0.75	none
1-Hexadecanol	C00823	1.12	0.17	0.4510	0.70	none
4-Oxoglutaramate	C05572	0.84	-0.25	0.4566	0.80	none
O-Phosphoethanolamine	C00346	1.45	0.53	0.4589	0.72	none
Cytosine	C00380	0.89	-0.17	0.4590	0.66	none
Nicotinic acid	C00253	2.62	1.39	0.4915	0.70	none
Thiabendazole	C07131	0.97	-0.05	0.4977	0.66	none
Pyroglutamic acid	C01879	0.81	-0.30	0.5006	0.66	none
AMP	C00020	0.87	-0.20	0.5079	0.58	none
Deoxycholic acid	C04483	0.91	-0.14	0.5087	0.64	none
Adenine	C00147	1.48	0.57	0.5220	0.64	none
Benzoate	C00180	1.29	0.37	0.5257	0.62	none
Maleic acid	C01384	1.16	0.21	0.5328	0.60	none
Ketoleucine	C00233	1.71	0.77	0.5336	0.60	none
Maltol	C11918	0.82	-0.29	0.5408	0.55	none
Quinolinic acid	C03722	0.85	-0.23	0.5427	0.61	none
Thiamine	C00378	1.08	0.11	0.5511	0.59	none
Fisetin	C10041	0.83	-0.27	0.5512	0.55	none
Beta-Carboline	C20157	1.02	0.03	0.5626	0.55	none

UDP	C00015	1.12	0.16	0.5736	0.50	none
Anabasine	C06180	1.02	0.03	0.5750	0.53	none
2-Keto-glutaramic acid	C00940	0.39	-1.37	0.5771	0.52	none
Dehydroascorbate	C05422	1.34	0.42	0.5840	0.57	none
2-Ketobutyric acid	C00109	1.48	0.56	0.5885	0.55	none
Erucic acid	C08316	1.69	0.76	0.5889	0.54	none
Glutathione	C00051	1.10	0.14	0.5903	0.54	none
Hydroquinone	C00530	1.86	0.90	0.5936	0.46	none
Citraconic acid	C02226	1.12	0.16	0.5976	0.53	none
Beta-Guanidinopropionic acid	C03065	0.65	-0.62	0.5982	0.47	none
Guanidinoacetate	C00581	1.06	0.09	0.5984	0.49	none
Sodium deoxycholate	C11171	0.69	-0.53	0.5993	0.49	none
3-Methyl-2-oxovaleric acid	C03465	1.71	0.77	0.6030	0.55	none
Dehydroepiandrosterone	C01227	1.11	0.15	0.6188	0.48	none
Phenylethylamine	C05332	1.26	0.33	0.6367	0.48	none
NAD	C00003	0.94	-0.09	0.6522	0.44	none
(S)-Methylmalonic acid semialdehyde	C06002	1.24	0.31	0.6546	0.45	none
Homovanillic acid	C05582	1.05	0.07	0.6639	0.42	none
alpha-Tocopherol	C02477	1.51	0.59	0.6650	0.38	none
1-Pyrroline-4-hydroxy-2-carboxylate	C04282	0.70	-0.51	0.6704	0.43	none
Succinic acid	C00042	0.60	-0.75	0.6852	0.42	none
Alpha-D-Glucose	C00267	0.62	-0.68	0.7001	0.40	none
1,2,3-Trihydroxybenzene	C01108	0.83	-0.27	0.7021	0.38	none
D-Mannose	C00159	1.09	0.12	0.7090	0.37	none
8-Amino-7-oxononanoate	C01092	0.97	-0.05	0.7098	0.40	none
2-Aminophenol	C01987	1.03	0.04	0.7368	0.29	none
4-Hydroxycinnamic acid	C00811	1.02	0.02	0.7398	0.36	none
Antibiotic JI-20A	C17704	1.53	0.61	0.7452	0.30	none
S-Adenosylmethionine	C00019	0.90	-0.15	0.7509	0.30	none
2,3-Butanediol	C00265	0.88	-0.18	0.7587	0.27	none
Pelargonic acid	C01601	1.05	0.08	0.7696	0.27	none
Diaminopimelic acid	C00666	0.97	-0.05	0.7701	0.31	none
Ethylmethylacetic acid	C18319	1.05	0.07	0.7739	0.31	none
Lacto-N-biose I	C06372	1.03	0.04	0.7825	0.21	none
3'-AMP	C01367	0.98	-0.03	0.7890	0.30	none
Acetylcysteine	C06809	1.18	0.24	0.7908	0.22	none
Pipecolic acid	C00408	1.23	0.30	0.7916	0.28	none
Imidazol-5-yl-pyruvate	C03277	0.99	-0.02	0.7925	0.28	none
L-Isoleucine	C00407	0.98	-0.03	0.7955	0.23	none
Phosphonoacetate	C05682	1.07	0.10	0.8031	0.22	none
L-Arginine	C00062	0.99	-0.02	0.8116	0.25	none
L-Proline	C00148	1.06	0.08	0.8141	0.25	none
Acetylcholine chloride	C08201	0.98	-0.04	0.8152	0.26	none
NADH	C00004	1.02	0.02	0.8169	0.22	none

Succinic acid semialdehyde	C00232	1.02	0.03	0.8251	0.22	none
Palmitic acid	C00249	1.02	0.03	0.8271	0.22	none
D-Ribose	C00121	0.87	-0.20	0.8287	0.19	none
4-Hydroxybenzaldehyde	C00633	1.01	0.01	0.8288	0.25	none
Cysteinylglycine	C01419	1.02	0.03	0.8320	0.19	none
Myristic acid	C06424	0.98	-0.03	0.8329	0.22	none
6-Hydroxynicotinic acid	C01020	1.01	0.02	0.8339	0.20	none
Pyrrole-2-carboxylic acid	C05942	1.20	0.27	0.8394	0.17	none
3-Carbamoyl-2-phenylpropionaldehyde	C16587	1.00	0.00	0.8410	0.21	none
1-Methyluric acid	C16359	0.98	-0.03	0.8428	0.24	none
Imidazole-4-acetaldehyde	C05130	1.10	0.14	0.8484	0.21	none
Pantothenol	C05944	0.89	-0.17	0.8503	0.15	none
trans-Cinnamate	C00423	1.16	0.21	0.8525	0.17	none
Citrulline	C00327	0.98	-0.03	0.8593	0.14	none
Glutaric acid	C00489	1.04	0.06	0.8612	0.21	none
Aminocaproic acid	C02378	0.97	-0.04	0.8765	0.18	none
O-Acetylserine	C00979	0.98	-0.02	0.8804	0.11	none
Cis-zeatin	C00371	1.11	0.15	0.8873	0.14	none
Benzylamine	C15562	0.92	-0.12	0.8961	0.16	none
Guanidinosuccinic acid	C03139	0.85	-0.23	0.9006	0.15	none
Adenosine diphosphate ribose	C00301	0.97	-0.04	0.9034	0.14	none
Isoliquiritigenin	C08650	0.71	-0.50	0.9040	0.11	none
Se-Methylselenocysteine	C05689	0.81	-0.31	0.9064	0.10	none
Uracil	C00106	0.97	-0.04	0.9072	0.13	none
Propionylcarnitine	C03017	1.03	0.04	0.9231	0.12	none
Gabapentin	C07018	1.00	0.00	0.9314	0.07	none
Methylmalonic acid	C02170	1.03	0.04	0.9378	0.06	none
Uridine	C00299	0.97	-0.04	0.9444	0.08	none
Stearic acid	C01530	0.99	-0.01	0.9488	0.07	none
D-Fructose	C00095	1.00	0.00	0.9541	0.05	none
Citric acid	C00158	1.10	0.13	0.9544	0.11	none
Pyridoxal 5'-phosphate	C00018	1.00	0.00	0.9578	0.03	none
Thymine	C00178	1.00	0.00	0.9624	0.07	none
Lipoxin A4	C06314	1.10	0.14	0.9635	0.02	none
alpha-D-Ribose 1-phosphate	C00620	1.15	0.21	0.9707	0.01	none
D-Glucose	C00031	0.98	-0.04	0.9727	0.05	none
1-Pyrroline-2-carboxylic acid	C03564	0.88	-0.18	0.9760	0.02	none
Heptanoic acid	C17714	1.12	0.17	0.9797	0.07	none
UMP	C00105	1.02	0.03	0.9825	0.06	none
5'-Methylthioadenosine	C00170	0.99	-0.02	0.9915	0.05	none
p-Aminobenzoic acid	C00568	1.01	0.01	0.9967	0.05	none
(2S)-Liquiritigenin	C09762	2.48	1.31	0.9985	0.04	none

Table S2. The results of KEGG enrichment analysis.

pathway_id	pathway_name	Total	Hits	*p*-value	-Log$_{10}$(*p*-value)	FDR	Impact
hsa04974	Protein digestion and absorption	47	8	0.0000	15.89	0.000	0.17
hsa00970	Aminoacyl-tRNA biosynthesis	52	8	0.0000	15.06	0.000	0.15
hsa05230	Central carbon metabolism in cancer	37	6	0.0000	11.81	0.001	0.11
hsa04978	Mineral absorption	29	5	0.0000	10.29	0.002	0.14
hsa02010	ABC transporters	138	8	0.0004	7.76	0.024	0.06
hsa00290	Valine, leucine and isoleucine biosynthesis	23	3	0.0034	5.68	0.157	0.19
hsa04150	mTOR signaling pathway	4	1	0.0531	2.93	1.000	0.25
hsa00780	Biotin metabolism	28	2	0.0543	2.91	1.000	0.22
hsa00770	Pantothenate and CoA biosynthesis	30	2	0.0615	2.79	1.000	0.07
hsa00410	beta-Alanine metabolism	32	2	0.0689	2.68	1.000	0.04
hsa04742	Taste transduction	32	2	0.0689	2.68	1.000	0.05
hsa00400	Phenylalanine, tyrosine and tryptophan biosynthesis	34	2	0.0766	2.57	1.000	0.11
hsa04916	Melanogenesis	6	1	0.0787	2.54	1.000	0.17
hsa00350	Tyrosine metabolism	78	3	0.0876	2.44	1.000	0.08
hsa05030	Cocaine addiction	7	1	0.0912	2.40	1.000	0.09
hsa04977	Vitamin digestion and absorption	39	2	0.0970	2.33	1.000	0.06
hsa00380	Tryptophan metabolism	83	3	0.1010	2.29	1.000	0.14
hsa05143	African trypanosomiasis	8	1	0.1035	2.27	1.000	0.13
hsa00280	Valine, leucine and isoleucine degradation	42	2	0.1100	2.21	1.000	0.03
hsa05031	Amphetamine addiction	9	1	0.1157	2.16	1.000	0.08
hsa05034	Alcoholism	10	1	0.1277	2.06	1.000	0.07
hsa04917	Prolactin signaling pathway	11	1	0.1395	1.97	1.000	0.05
hsa04540	Gap junction	11	1	0.1395	1.97	1.000	0.09
hsa05231	Choline metabolism in cancer	11	1	0.1395	1.97	1.000	0.11
hsa00260	Glycine, serine and threonine metabolism	50	2	0.1464	1.92	1.000	0.04
hsa04728	Dopaminergic synapse	12	1	0.1512	1.89	1.000	0.05
hsa04721	Synaptic vesicle cycle	12	1	0.1512	1.89	1.000	0.08
hsa05131	Shigellosis	14	1	0.1742	1.75	1.000	0.05
hsa00360	Phenylalanine metabolism	60	2	0.1947	1.64	1.000	0.07
hsa05012	Parkinson disease	21	1	0.2498	1.39	1.000	0.03
hsa00220	Arginine biosynthesis	23	1	0.2701	1.31	1.000	0.03
hsa04714	Thermogenesis	23	1	0.2701	1.31	1.000	0.05

hsa00565	Ether lipid metabolism	25	1	0.2899	1.24	1.000	0.02
hsa04024	cAMP signaling pathway	25	1	0.2899	1.24	1.000	0.04
hsa04216	Ferroptosis	29	1	0.3279	1.12	1.000	0.09
hsa00730	Thiamine metabolism	31	1	0.3461	1.06	1.000	0.02
hsa00230	Purine metabolism	95	2	0.3708	0.99	1.000	0.06
hsa04976	Bile secretion	97	2	0.3806	0.97	1.000	0.02
hsa04750	Inflammatory mediator regulation of TRP channels	35	1	0.3812	0.96	1.000	0.03
hsa00480	Glutathione metabolism	38	1	0.4063	0.90	1.000	0.03
hsa04726	Serotonergic synapse	42	1	0.4382	0.83	1.000	0.03
hsa00340	Histidine metabolism	47	1	0.4757	0.74	1.000	0.08
hsa00640	Propanoate metabolism	48	1	0.4829	0.73	1.000	0.02
hsa00310	Lysine degradation	50	1	0.4970	0.70	1.000	0.03
hsa04080	Neuroactive ligand-receptor interaction	52	1	0.5108	0.67	1.000	0.02
hsa00564	Glycerophospholipid metabolism	52	1	0.5108	0.67	1.000	0.03
hsa00983	Drug metabolism - other enzymes	52	1	0.5108	0.67	1.000	0.04
hsa00061	Fatty acid biosynthesis	58	1	0.5499	0.60	1.000	0.01
hsa00270	Cysteine and methionine metabolism	63	1	0.5801	0.54	1.000	0.05
hsa00240	Pyrimidine metabolism	65	1	0.5916	0.52	1.000	0.00
hsa01040	Biosynthesis of unsaturated fatty acids	74	1	0.6397	0.45	1.000	0.02
hsa00330	Arginine and proline metabolism	78	1	0.6593	0.42	1.000	0.04
hsa00130	Ubiquinone and other terpenoid-quinone biosynthesis	92	1	0.7199	0.33	1.000	0.01
hsa00140	Steroid hormone biosynthesis	99	1	0.7462	0.29	1.000	0.00
hsa00860	Porphyrin and chlorophyll metabolism	142	1	0.8619	0.15	1.000	0.00

Table S3. The gradient elution procedure of liquid chromatography in ESI+ mode.

Time (min)	A (%)	B (%)
0-1	2	98
1-9	2-50	98-50
9-12	50-98	50-2
12-13.5	98	2
13.5-14	98-2	2-98
14-20	2	98

Table S4. The gradient elution procedure of liquid chromatography ESI- mode.

Time (min)	C (%)	D (%)
0-1	2	98
1-9	2-50	98-50
9-12	50-98	50-2
12-13.5	98	2
13.5-14	98-2	2-98
14-17	2	98

Table S5. Mass spectrum conditions.

Item	Parameter
Sheath gas pressure	30 arb
Aux gas flow	10 arb
Spray voltage	ESI+: 3.50 kV<br>ESI-: -2.50 kV
Capillary temperature	325 °C
MS1 range	m/z 100-1000
MS1 resolving power	60000 FWHM
MS/MS resolving power	15000 FWHM
Number of data dependent scans per cycle	4
Normalized collision energy	30%
Dynamic exclusion time	automatic
